# Patients undergoing surgery for lumbar spinal stenosis experience unique courses of pain and disability: A group-based trajectory analysis

**DOI:** 10.1371/journal.pone.0224200

**Published:** 2019-11-07

**Authors:** Jeffrey J. Hebert, Edward Abraham, Niels Wedderkopp, Erin Bigney, Eden Richardson, Mariah Darling, Hamilton Hall, Charles G. Fisher, Y. Raja Rampersaud, Kenneth C. Thomas, Bradley Jacobs, Michael Johnson, Jerome Paquet, Najmedden Attabib, Peter Jarzem, Eugene K. Wai, Parham Rasoulinejad, Henry Ahn, Andrew Nataraj, Alexandra Stratton, Neil Manson

**Affiliations:** 1 Faculty of Kinesiology, University of New Brunswick, Fredericton, Canada; 2 School of Psychology and Exercise Science, Murdoch University, Perth, Australia; 3 Canada East Spine Centre, Saint John, New Brunswick, Canada; 4 Division of Orthopaedic Surgery, Zone 2, Horizon Health Network, Saint John, New Brunswick, Canada; 5 Dalhousie University Faculty of Medicine, Halifax, Nova Scotia, Canada; 6 Department of Regional Health Research, University of Southern Denmark, Odense, Denmark; 7 The Orthopedic Department, Hospital of Southwestern Jutland, Esbjerg, Denmark; 8 University of Toronto, Department of Surgery, Toronto, Canada; 9 Combined Neurosurgical and Orthopedic Spine Program, Department of Orthopedic Surgery, University of British Columbia, Blusson Spinal Cord Centre, Vancouver, British Columbia, Canada; 10 University of Toronto, University Health Network, Arthritis Program, Krembil Research Institute, Toronto, Ontario, Canada; 11 University of Calgary, Foothills Medical Centre, Calgary, Alberta, Canada; 12 Department of Clinical Neurosciences, Division of Neurosurgery-Spine Program, University of Calgary, Calgary, Alberta, Canada; 13 Department of Surgery, Section of Orthopedics and Neurosurgery, University of Manitoba, Winnipeg, Manitoba, Canada; 14 Division of Neurosurgery, Department of Surgery, CHU de Quebec-Universite Laval, Quebec City, Quebec, Canada; 15 Division of Neurosurgery, Zone 2, Horizon Health Network, Saint John, New Brunswick, Canada; 16 McGill Scoliosis and Spine Research Group, Montreal, Quebec, Canada; 17 Division of Orthopaedics, McGill University Health Centre, Montreal, Quebec, Canada; 18 Department of Surgery, University of Ottawa, Ottawa, Ontario, Canada; 19 London Health Sciences Center, Victoria Hospital, London, Ontario, Canada; 20 Division of Orthopaedic Surgery, Department of Surgery, Schulich School of Medicine, Western University, London, Ontario, Canada; 21 University of Toronto Spine Program, Toronto, Ontario, Canada; 22 Division of Neurosurgery, Department of Surgery, Faculty of Medicine and Dentistry, University of Alberta Hospital, Edmonton, Alberta, Canada; Universita degli Studi di Palermo, ITALY

## Abstract

**Objective:**

Identify patient subgroups defined by trajectories of pain and disability following surgery for degenerative lumbar spinal stenosis, and investigate the construct validity of the subgroups by evaluating for meaningful differences in clinical outcomes.

**Methods:**

We recruited patients with degenerative lumbar spinal stenosis from 13 surgical spine centers who were deemed to be surgical candidates. Study outcomes (leg and back pain numeric rating scales, modified Oswestry disability index) were measured before surgery, and after 3, 12, and 24 months. Group-based trajectory models were developed to identify trajectory subgroups for leg pain, back pain, and pain-related disability. We examined for differences in the proportion of patients achieving minimum clinically important change in pain and disability (30%) and clinical success (50% reduction in disability or Oswestry score ≤22) 12 months from surgery.

**Results:**

Data from 548 patients (mean[SD] age = 66.7[9.1] years; 46% female) were included. The models estimated 3 unique trajectories for leg pain (excellent outcome = 14.4%, good outcome = 49.5%, poor outcome = 36.1%), back pain (excellent outcome = 13.1%, good outcome = 45.0%, poor outcome = 41.9%), and disability (excellent outcome = 30.8%, fair outcome = 40.1%, poor outcome = 29.1%). The construct validity of the trajectory subgroups was confirmed by between-trajectory group differences in the proportion of patients meeting thresholds for minimum clinically important change and clinical success after 12 postoperative months (*p* < .001).

**Conclusion:**

Subgroups of patients with degenerative lumbar spinal stenosis can be identified by their trajectories of pain and disability following surgery. Although most patients experienced important reductions in pain and disability, 29% to 42% of patients were classified as members of an outcome trajectory subgroup that experienced little to no benefit from surgery. These findings may inform appropriate expectation setting for patients and clinicians and highlight the need for better methods of treatment selection for patients with degenerative lumbar spinal stenosis.

## Introduction

Degenerative lumbar spinal stenosis (LSS) is a common musculoskeletal disorder, experienced by 1 in 5 adults 65 years or older [1, 2]. Although there are no standardized diagnostic criteria[3], LSS is typically characterized by neurogenic claudication and functional limitations [4].

Surgery for LSS is the most frequent type of spinal surgery performed on older adults and will become increasingly common as the population ages [5]. Consistent with the clinical experiences of many spine surgeons, early clinical evidence for the efficacy [6, 7] and cost-effectiveness [8] of surgery for LSS is promising. However, challenges in carrying out randomized trials in surgery persist, as do issues around generalizability and patient selection [3, 9]. Clinicians treating patients with LSS would benefit from expanded evidence to guide decisions around operative and non-operative interventions.

A recent systematic review and meta-regression of data from 39 (pain outcome) and 31 (disability outcome) primary studies identified the average postoperative outcome trajectories experienced by patients with LSS [10]. The average patient experienced substantial improvements in pain and disability within 3 postoperative months, and these improvements were stable over the subsequent 5 years. However, some outcome studies reported that patients experienced a near resolution of symptoms, while others reported little change. This heterogeneity in outcomes suggests that many patients may not fit the average trajectory profiles for pain and disability [10].

No prior studies have attempted to identify outcome trajectory subgroups following surgery for LSS. This knowledge can further our understanding of the prognosis and outcomes experienced by these patients. The aims of this study were to 1) identify patient subgroups defined by trajectories of pain and disability following surgery for LSS, and 2) investigate the construct validity of the trajectory subgroups by evaluating for meaningful differences in clinical outcomes.

## Methods

### Study design and participants

This study was a retrospective analysis of prospectively collected data from patients enrolled in the Canadian Spine Outcomes and Research Network (CSORN), a multicenter initiative of orthopaedic and neurological spine surgeons. The CSORN includes a surgical registry comprising preoperative baseline and postoperative follow-up data to document the clinical outcomes experienced by patients undergoing spine surgery. The accuracy and completeness of registry information is monitored and audited by a dedicated Data Quality Coordinator. Clinical outcome data were collected at the preoperative baseline and after 3, 12, and 24 months postoperatively.

In the current study, we included data from all patients 50 years and older with a chief pathology of lumbar spinal stenosis, as identified by the consulting spine surgeon. All patients underwent surgery for decompression at one or more spinal levels with or without fusion. The CSORN project was originally approved by Research Ethics Boards local to each data collection site. Prior to study enrolment, all patients reviewed information about the study and provided written informed consent by signing forms approved by the relevant Research Ethics Board. The current study protocol was approved by Research Ethics Boards of the Horizon Health Network (2017–2568) and University of New Brunswick (2018–025).

### Demographic and clinical information

After obtaining consent, preoperative data collection included demographic (age, sex) and clinical information (body mass index, smoking history, comorbidities and history of spinal surgery). The attending spine surgeon and surgical staff recorded the surgical details including type of surgery, number of spinal levels and the use of minimally invasive techniques.

### Clinical outcomes

#### Leg and back pain intensity

Leg pain intensity and back pain intensity were measured separately with 11-point numeric pain rating scales [11]. Pain ratings represented the typical pain experienced over the preceding 24 hours, with potential scores ranging from 0 (‘no pain’) to 10 (‘worst pain imaginable’). Ratings can be categorized as ‘mild’ (0–3), ‘moderate (4–6), or ‘severe’ (7–10) [12, 13]. The numeric pain rating scale has excellent test-retest reliability and responsiveness [14, 15]. The minimum level of important change for patients with back pain is estimated to be 30% [16].

#### Pain-related disability

The modified Oswestry disability questionnaire was used to quantify disability related to leg and back pain [17]. Patients rated the difficulty of 10 functional activities (e.g., walking, lifting) yielding total scores from 0 to 100, with higher values indicating greater disability. Total scores are classified as: 0–20 ‘minimal disability’, 21–40 ‘moderate disability’, 41–60 ‘severe disability’, 61–80 ‘crippled’, and 81–100 ‘bed-bound or exaggerating’. This questionnaire has excellent test-retest reliability and responsiveness [17], and an estimated minimum level of important change of 30% [16]. Previous research has reported relative and absolute measures of clinical success to be at least 50% improvement for patients undergoing non-operative therapy [18], and achieving a score of ≤22 for patients receiving surgery for degenerative lumbar spine disorders [19].

### Data analysis

All analyses were conducted with Stata 15.1 software (StataCorp, College Station, TX, USA). Pain and disability outcomes at preoperative baseline and 3, 12, and 24 months after surgery were used to assign each patient to a trajectory group using group-based trajectory modeling. Compared to variable-centered analyses that seek to describe associations between variables (e.g., regression), person-centered approaches such as group-based trajectory modeling identify groups of individuals who share particular attributes (e.g., course of symptoms over time).[20] This approach is a specialized application of finite mixture modeling that provides an empirical method of identifying meaningful subgroups of patients, based on their patterns of change (i.e., trajectories) in outcome over time [21, 22]. Unlike growth mixture modeling, group-based trajectory models use maximum likelihood estimation to approximate an unknown distribution of trajectories and do not assume that latent classes represent distinct populations [21]. Group-based trajectory models do not require the inclusion of additional covariates as unspecified models are not prone to misspecification like other trajectory modeling approaches [23]. Therefore, group-based trajectory models are well-suited to identify meaningful but unknown homogeneous subgroups (i.e., classes) that follow distinct trajectories, such as occurs with different clinical outcomes experienced by patients undergoing the same treatment.

Separate group-based trajectory models were created to identify trajectory subgroups for each outcome variable (leg pain intensity, back pain intensity, back and leg pain-related disability) applying a censored normal distribution. We excluded patients with missing outcome scores at baseline and those with less than two follow-up outcome measures. Group-based trajectory models handle missing data with maximum likelihood estimation, resulting in asymptotically unbiased parameter estimates when data are missing at random [21].

Some patients with degenerative lumbar stenosis have predominant leg pain, some have predominant back pain, and some have a surgical indication other than pain (e.g., deformity, motor deficit). Given the nature of the outcome measures, we excluded patients with minimal pain or disability at the preoperative baseline to help isolate clinically-relevant symptoms or impairments and permit judgements of clinically-important change. When modeling leg or back pain intensity, we excluded patients with baseline pain intensity scores less than 3 out of 10. When modeling disability, we excluded patients with baseline Oswestry scores ≤20 (i.e., minimal disability).

Initially, single class quadratic models were constructed and the number of classes were increased until optimal models were identified. Judgments regarding optimal model specification cannot be reduced to a single metric [21]. Our modeling decisions were based on a combination of statistical and clinical judgments. We first used the Bayesian information criterion to identify optimal model fit with minimum class sizes of 5%, and subsequently evaluated model outcomes to ensure that the trajectory classes were clinically relevant. Pain and disability trajectory classes were described using clinical judgement and categorical descriptors for the numeric rating scale and the Oswestry index. For example, minimal postoperative pain or disability was considered to represent an excellent outcome, while minimal improvement (i.e., persistent moderate-to severe pain or disability) was considered a poor outcome.

The final models were subsequently evaluated with 4 *a priori* diagnostic criteria: 1) a minimum average posterior probability of individual group membership of 0.7 for each group; 2) obtaining close correspondence between the estimated probability of group membership and the proportion of participants assigned to each group based on the posterior probability; 3) reasonably tight confidence intervals around estimated group membership probabilities (calculated with the Stata command for nonlinear combinations of parameter estimates) and 4) minimum odds of correct classification >5 [21, 22]. We tested the internal validity of the final models by replicating the modeling procedures by random split-half sampling.

To explore the construct validity of the leg pain, back pain, and disability trajectory groups, we generated descriptive statistics for each pain and disability outcome, stratified by trajectory class. Additionally, we calculated the proportion of patients within each trajectory who met 5 clinical benchmarks at the 12-month follow-up: minimum clinically important change (30%) in 1) leg pain, 2) back pain, and 3) disability, as well as 4) relative (50% improvement) and 5) absolute (≤ 22) estimates of successful disability outcome. Differences between trajectory groups in the proportion of patients meeting each clinical benchmark were examined with Fischer’s exact test. Alpha was .05 for all analyses.

## Results

**Fig 1:**
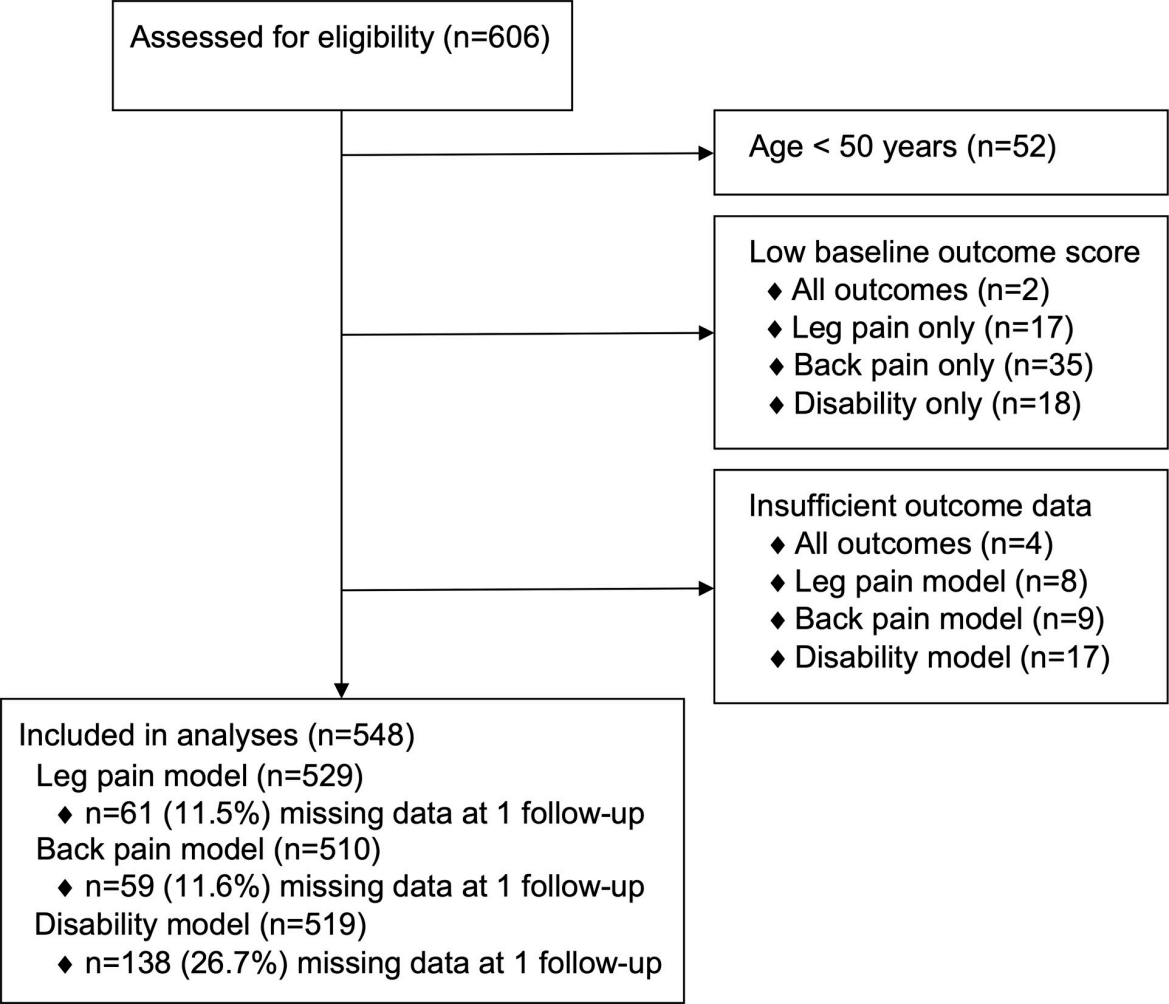
Study flow diagram.

Data from 606 patients were assessed for eligibility. After applying our selection and analysis criteria, we excluded data from 58 participants (n = 52 age <50; n = 6 insufficient outcome data). In total, data from 548 patients who underwent surgery performed by one of 34 surgeons from 13 sites were included in one or more analysis (Fig 1). Preoperative demographic, clinical, and surgical information for patients who were included in the analysis and excluded due to low preoperative pain or disability or insufficient outcome data are presented in Table 1.

**Table 1:** Baseline characteristics and surgical details of patients included in the analysis or excluded due to low preoperative pain or disability or insufficient outcome data ^1^.

Variable	Patients included in analysis (n = 548)	Patients excluded from analysis (n = 6)^2^
Age [mean ± SD]	66.7 ± 9.1	72 ± 11.2
Female sex	250 (45.6%)	0 (0.0%)
Body mass index [mean ± SD]	28.2 ± 8.4	29.4 ± 6.0
Smoking status		
Non-smoker	433 (79.0%)	3 (50.0%)
Current smoker	83 (15.2%)	0 (0.0%)
Previous smoker	22 (4.0%)	0 (0.0%)
Missing/elected not to answer	10 (1.8%)	3 (50.0%)
Previous spine surgery		
Yes	137 (25.0%)	0 (0.0%)
No	398 (72.6%)	3 (50.0%)
Missing	13 (2.4%)	3 (50.0%)
Number of comorbidities		
0	139 (25.4%)	5 (83.3%)
1	134 (24.5%)	1 (16.7%)
2	94 (17.2%)	0 (0.0%)
3	75 (13.7%)	0 (0.0%)
>3	106 (19.3%)	0 (0.0%)
Surgery type		
Decompression	244 (44.5%)	3 (50.0%)
Decompression with fusion	301 (54.9%)	3 (50.0%)
Missing	3 (0.6%)	0 (0.0%)
Surgery number of spinal levels		
1	256 (46.7%)	4 (66.7%)
2	149 (27.2%)	0 (0.0%)
3	70 (12.8%)	1 (16.7%)
>3	69 (14.4%)	1 (16.7%)
Missing	4 (0.7%)	0 (0.0%)
Minimally invasive surgery	178 (32.5%)	4 (66.7%)

^1^ Values are number (percentage) unless otherwise indicated.

^2^ Reasons for exclusion: low preoperative pain or disability, insufficient outcome data.

**Fig 2:**
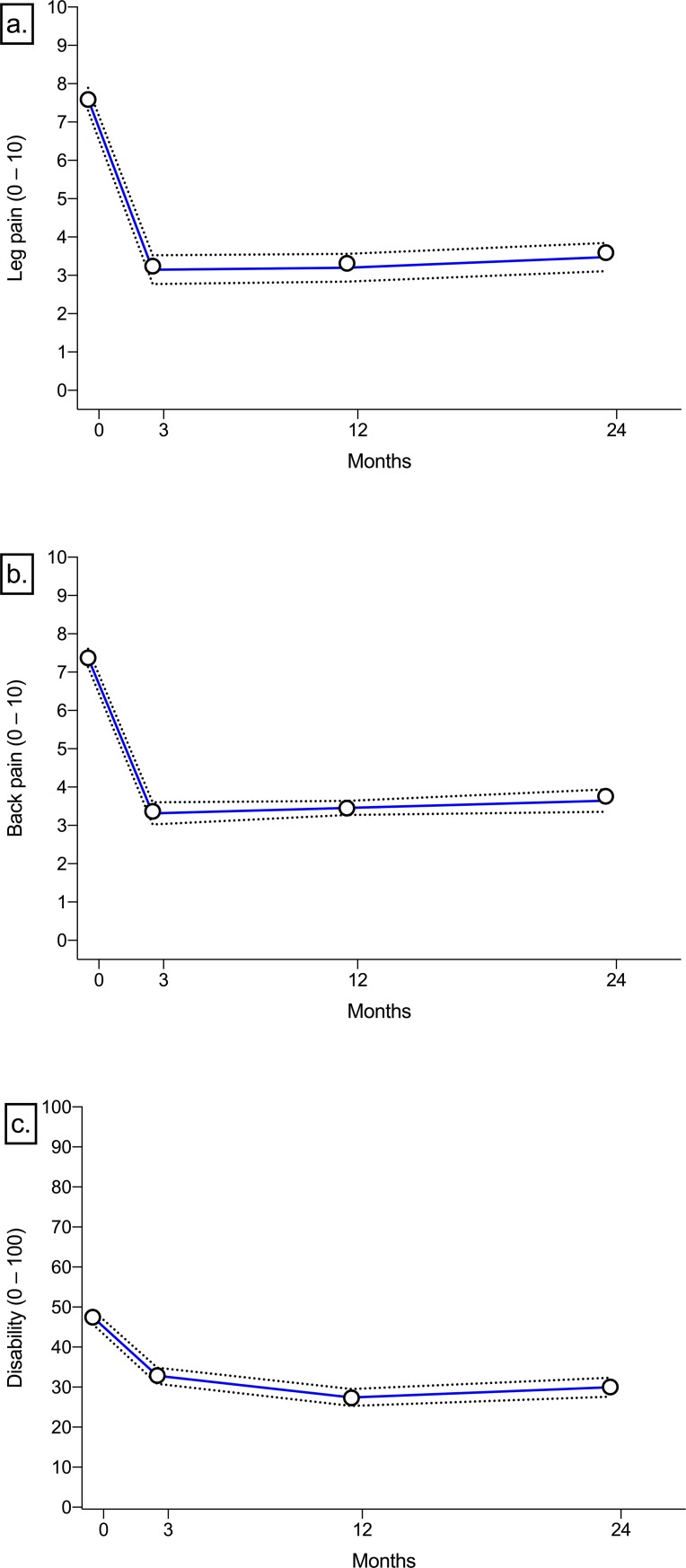
Average trajectories for a. leg pain, b. back pain, and c. disability. Point estimates are average outcome scores. Dotted lines represent 95% confidence intervals.

All final models identified clinically relevant 3-class solutions based on the Bayesian information criterion (S1 Table), and achieved adequate performance according to our predefined criteria (Table 2). Split-half sampling resulted in 3-class solutions with very similar trajectory classes and prevalence estimates for all outcomes (S1 Fig). Average clinical outcomes, stratified by trajectory group, are presented in Table 3, and average trajectories of leg pain, back pain, and disability are presented in Fig 2A–2C. Statistically significant (*p* < .001) differences between trajectory groups in the proportion of patients who met each of the 5 clinical benchmarks were identified (Table 4).

**Table 2:** Group-based trajectory diagnostics for leg pain, low back pain, and disability models.

	Average posterior probability^1^	Estimated membership % (95% CI)	Assigned membership %	Odds of correct classification^2^
**Leg pain trajectory groups (N = 529)**				
1, ‘excellent’	.87	14.4 (9.3 to 19.4)	14.2	39.50
2, ‘good’	.88	49.5 (43.3 to 55.8)	50.3	7.02
3, ‘poor’	.90	36.1 (29.7 to 42.4)	35.5	16.17
**Back pain trajectory groups (N = 510)**				
1, ‘excellent’	.85	13.1 (8.0 to 18.1)	12.8	37.62
2, ‘good’	.88	45.0 (39.1 to 50.9)	45.7	8.92
3, ‘poor’	.93	41.9 (36.4 to 47.5)	41.6	19.85
**Disability trajectory groups (N = 519)**				
1, ‘excellent’	.88	30.8 (24.1 to 37.6)	31.2	16.51
2, ‘fair’	.85	40.1 (33.9 to 46.3)	39.5	8.66
3, ‘poor’	.90	29.1 (23.4 to 34.8)	29.3	22.03

^1^: minimum threshold = .70

^2^: minimum threshold = 5.0

**Table 3:** Descriptive clinical outcomes stratified by trajectory group.

	Preoperative	3 months	12 months	24 months
**Leg pain trajectory groups (leg NRS score)**				
1, ‘excellent’	7.7 ± 1.6	.1 ± .3	.1 ± .3	.3 ± .7
2, ‘good–gradual’	7.4 ± 1.7	2.6 ± 2.3	2.3 ± 2.2	2.4 ± 2.1
3, ‘poor’	7.8 ± 1.6	5.4 ± 2.5	6.1 ± 2.1	6.6 ± 1.9
**Back pain trajectory groups (back NRS score)**				
1, ‘excellent’	7.5 ± 1.7	.4 ± .7	.3 ± 7	.3 ± .7
2, ‘good’	6.9 ± 1.8	2.4 ± 1.7	2.4 ± 1.6	2.4 ± 1.7
3, ‘poor’	7.9 ± 1.3	5.2 ± 2.0	5.6 ± 1.9	6.3 ± 1.6
**Disability trajectory groups (ODI score)**				
1, ‘excellent’	40.8 ± 11.3	14.0 ± 10.9	8.0 ± 6.6	10.8 ± 9.4
2, ‘fair’	45.5 ± 11.4	33.9 ± 13.1	26.4 ± 11.9	30.3 ± 11.8
3, ‘poor’	57.2 ± 11.5	51.7 ± 12.1	49.3 ± 11.1	51.0 ± 10.5

Values are mean ± SD

NRS = numeric rating scale; ODI = modified Oswestry disability index

**Table 4:** Proportion of patients meeting 12-month clinical outcome benchmarks, stratified by trajectory group.

Excellent outcome	Leg pain MCIC^1^	Back pain MCIC^2^	ODI MCIC^3^	Relative ODI success^4^	Absolute ODI success^5^
**Leg pain trajectory groups**					
1, ‘excellent’	100%	97.3%	93.2%	87.7%	86.7%
2, ‘good’	89.0%	79.2%	72.8%	56.4%	54.2%
3, ‘poor’	37.0%	51.4%	33.9%	18.6%	20.4%
**Back pain trajectory groups**					
1, ‘excellent’	90.6%	100%	84.4%	80.0%	80.0%
2, ‘good’	81.3%	88.8%	77.0%	62.8%	59.2%
3, ‘poor’	55.5%	44.2%	35.6%	16.6%	18.7%
**Disability trajectory groups**					
1, ‘excellent’	91.8%	93.1%	96.3%	93.2%	97.5%
2, ‘fair’	69.0%	69.5%	62.8%	41.7%	40.7%
3, ‘poor’	54.1%	49.0%	22.0%	5.3%	0.7%

^1^: ≥30% reduction in NRS for leg pain

^2^: ≥30% reduction in NRS for back pain

^3^: ≥30% reduction in ODI

^4^: ≥50% reduction in ODI

^5^: ODI score ≤22

Note: between-group differences in the proportion of patients meeting each of the clinical benchmarks were statistically significant (*p* < .001).

Green ≥75%; yellow 50–74%; red <50%

MCIC = minimum clinically important change; ODI = modified Oswestry disability index; NRS = numeric rating scale

### Leg pain trajectories

**Fig 3:**
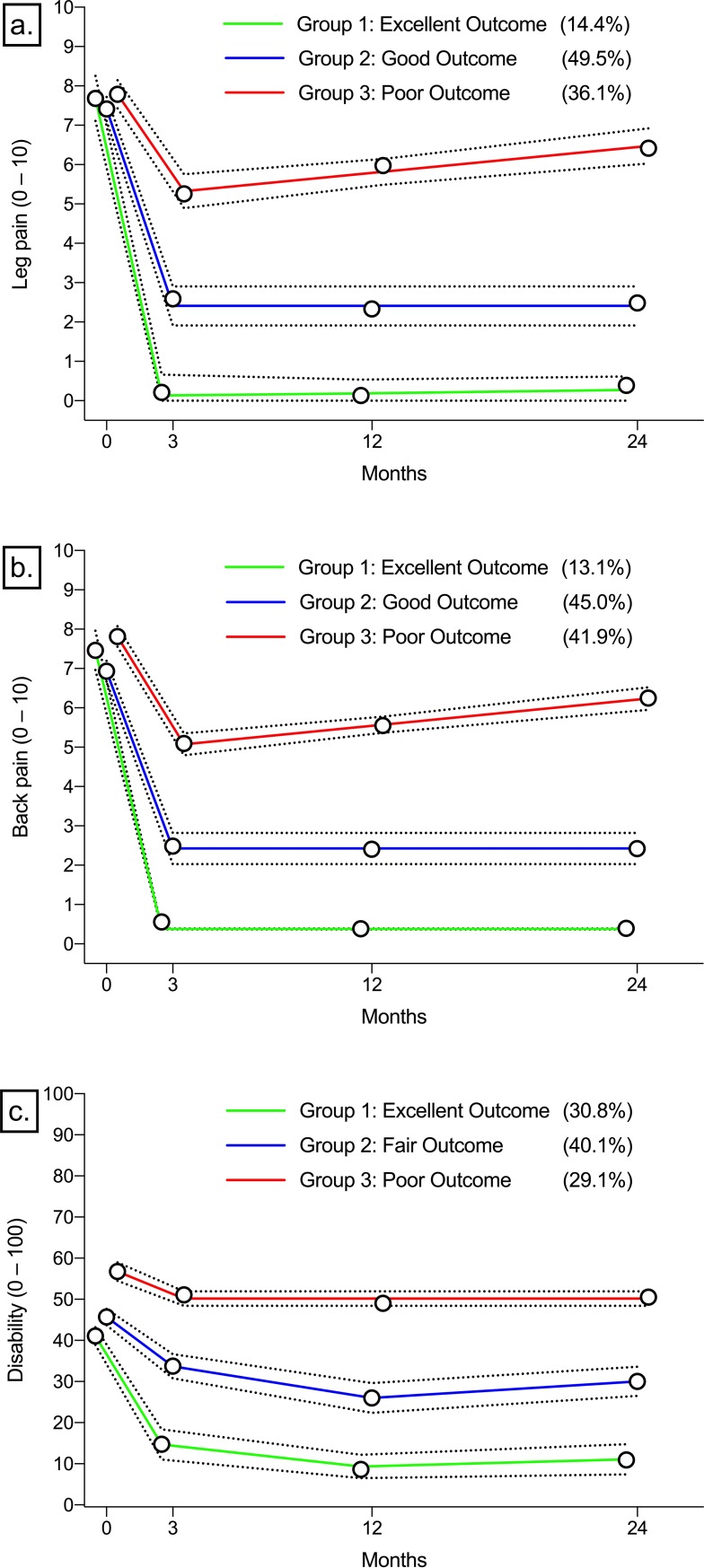
Clinical outcome trajectory groups with prevalence estimates. a. leg pain trajectories (N = 529); b. low back pain trajectories (N = 510); c. disability trajectories (N = 519). Point estimates are average outcome scores (0–10 numeric pain rating scale or 0–100 modified Oswestry index). Dotted lines represent 95% confidence intervals.

The leg pain trajectory model identified 3 distinct trajectory groups (Fig 3A). Group 1 (excellent outcome) comprised 14.4% of patients who experienced large improvements in leg pain intensity, with nearly no pain by 3 months and a stable course thereafter. All patients in Group 1 experienced a clinically important change in their leg pain, and nearly all (86.7% to 87.7%) achieved a successful disability outcome at 12 months (Table 4). Patients in Group 2 (49.5%, good outcome) experienced improved but persistent mild leg pain that continued over the course of follow-up. Finally, 36.1% of patients were categorized in Group 3 (poor outcome). These patients experienced minimal improvement at 3 months, and mild regression toward preoperative levels of leg pain 12 and 24 months from surgery. Only 1 in 3 patients (37.0%) in Group 3 reported a clinically important change in their leg pain, with about 18.6% to 20.4% of patients reporting a successful disability outcome after 12 postoperative months (Table 4).

### Back pain trajectories

Three trajectory groups were identified by the back pain trajectory model (Fig 3B). Group 1 (excellent outcome) included 13.1% of patients who experienced large improvements in back pain intensity, and nearly no pain after 3 months. All patients in Group 1 experienced clinically important reductions in back pain and nearly all patients experienced successful 12-month disability outcomes (80.0%) (Table 4). Group 2 (good outcome) included 45.0% of patients with moderately improved but persistent mild back pain that continued over the course of follow-up. Patients in Group 3 (poor outcome) comprised 41.9% of patients who experienced short-term improvements in back pain intensity and mild worsening of pain at 12 and 24 months. Only 44.2% of patients from Group 3 achieved clinically important change in back pain by 12 months with less than 1 in 5 patients (16.6% to 18.7%) experiencing a successful disability outcome (Table 4).

### Pain-related disability

The disability trajectory model identified 3 unique trajectory groups (Fig 3C). Group 1 (excellent outcome) included 30.8% of patients who reported rapid improvements in disability, with nearly all patients experiencing clinically important changes in disability (91.8%) and successful outcomes (93.2% to 97.5%) after 12 months (Table 4). Group 2 (fair outcome) included 40.1% of patients with modest improvement in disability, less than half of whom (40.7% to 41.7%) experienced a successful clinical outcome at 12 months (Table 4). Patients in Group 3 (poor outcome) included 29.1% of patients with minimal improvement following surgery and who continued to experience severe, persistent disability. Approximately than 1 in 5 patients in Group 3 (22.0%) achieved clinically important changes in disability, with very few patients (0.7% to 5.3%) experiencing a successful clinical outcome at 12 months (Table 4).

## Discussion

We present the first evidence of pain and disability trajectory subgroups among surgically treated patients with LSS. Unique trajectories of leg pain, back pain, and pain-related disability were identified and the construct validity of these subgroups was confirmed through differences in the proportion of patients meeting thresholds for minimum clinically-important change and clinical success. The trajectory subgroups represent potentially useful pain and disability phenotypes as their identification advances knowledge regarding prognosis and the clinical outcomes experienced by patients with LSS. Although most patients in this study experienced substantial reductions in pain and disability following surgery, 29% to 42% of patients (depending on outcome) were classified as members of a pain or disability trajectory group that experienced little or no benefit from surgery, underscoring the need for better methods of patient selection.

The average pain and disability trajectories identified in our study were very similar to a recent systematic review and meta-analysis reporting the average pain and pain-related disability experienced by patients undergoing surgery for LSS [10]. However, we also identified clinically-important subgroups demonstrating that most patients followed a pain or disability trajectory that differed from the average course. Compared to pain trajectories, disability trajectories appear more dependent on baseline score, suggesting that pain may be a more modifiable than pain-related disability following LSS surgery.

Shared decision making is an essential component of patient-centered care that promotes interaction between informed, engaged patients and clinicians to make optimal healthcare decisions [24]. High-quality clinical evidence is central to this process as shared decision-making is most useful in the context of ‘preference-sensitive’ therapeutic options (i.e., when best evidence demonstrates more than one acceptable treatment for a particular condition). Evidence demonstrating superiority for a specific surgical technique [9], or between surgical and conservative treatment options [3] is lacking, highlighting the appropriateness of shared decision-making between patients with LSS when selecting therapies. The current study results can inform shared-decision making by illustrating the different postoperative outcome trajectories typically experienced by patients, as well as the prevalence of each trajectory type. This knowledge can help patients and clinicians set appropriate expectations prior to LSS surgery.

There are strengths and limitations of this study. The primary study strengths include the large sample of patients from 13 spine centers and the long-term follow-up with patient-centered clinical outcomes of pain and disability. Additionally, we implemented a novel statistical technique to understand complex longitudinal data resulting in the identification of previously unknown pain and disability phenotypes. Although our study was conducted in Canadian spine centers, and the surgical outcomes experienced by patients in other countries may differ, the average back and leg pain outcomes experienced by patients in the current study were similar to those reported in a systematic review comprising outcome reports from 22 countries [10]. This speaks to the external validity of our results and supports their generalizability to patients in other jurisdictions. Although group-based trajectory models assume data to be missing at random, non-randomly missing data is a potential source of bias. All patients in this study had preoperative baseline data and follow-up data at 2 of 3 time points, and most patients (73% to 89%) had complete data available. We believe it unlikely that missing data had an important effect on the study results.

The use of trajectory modeling in spine surgery can advance our understanding of the outcomes experienced by patients, and help to identify clinically-relevant subgroups of patients who are more or less likely to respond to surgery. Significant clinical utility would be gained if the trajectory subgroups identified in this study could be accurately predicted prior to surgery, as this knowledge would enhance patient selection. It will also be important to determine whether membership in a particular trajectory group could be modifiable. For example, little is known about the effectiveness of postoperative care following spine surgery [25, 26] and optimal rehabilitation might shift the patient to a more beneficial trajectory. Therefore, identifying the preoperative predictors of trajectory group membership and the effect of postoperative care on pain and disability trajectories for patients with LSS will be important priorities for future research. While the former can be accomplished using high-quality longitudinal data and observational designs, the latter will require robust randomized clinical trials to identify the treatment effects resulting from postoperative care.

In conclusion, the current study identified subgroups of patients with LSS who can be identified by their trajectories of pain and disability following surgery. The construct validity of these subgroups was confirmed, and they may represent useful patient phenotypes. Although most patients experienced important reductions in pain and disability, many patients (29% to 42% depending on outcome) were classified as members of an outcome trajectory subgroup that experienced little to no benefit from surgery. These findings highlight the need for better methods of treatment selection for patients with LSS.

## Supporting information

S1 Table Model selection results according to Bayesian information criterion and smallest group size^1^. Bayesian information criterion value closest to zero indicates best model fit ^2^ Minimum acceptable size at least 5% ^3^ Bayesian information criterion (for the total number of participants) ^4^ Bayesian information criterion (for the total number of observations).
(DOCX)

S1 Fig Split sample clinical outcome trajectory groups with prevalence estimates. a1. sample 1 leg pain trajectories (N = 264), a2. sample 2 leg pain trajectories (N = 265); b1. sample 1 low back pain trajectories (N = 255), sample 2 low back pain trajectories (N = 255); c1. sample 1 disability trajectories (N = 259), c2. sample 2 disability trajectories (N = 260). Point estimates are average outcome scores (0–10 numeric pain rating scale or 0–100 modified Oswestry index). Dotted lines represent 95% confidence intervals.
(DOCX)

## References

[1] Otani K, Kikuchi S, Yabuki S, Igarashi T, Nikaido T, Watanabe K, et al Lumbar spinal stenosis has a negative impact on quality of life compared with other comorbidities: an epidemiological cross-sectional study of 1862 community-dwelling individuals. ScientificWorldJournal. 2013;2013:590652 10.1155/2013/590652 24453878 PMC3885225

[2] Ishimoto Y, Yoshimura N, Muraki S, Yamada H, Nagata K, Hashizume H, et al Associations between radiographic lumbar spinal stenosis and clinical symptoms in the general population: the Wakayama Spine Study. Osteoarthritis Cartilage. 2013;21(6):783–8. 10.1016/j.joca.2013.02.656 . 23473979

[3] Zaina F, Tomkins-Lane C, Carragee E, Negrini S. Surgical versus non-surgical treatment for lumbar spinal stenosis. Cochrane Database Syst Rev. 2016;(1):CD010264 10.1002/14651858.CD010264.pub2 . 26824399 PMC6669253

[4] Comer CM, Redmond AC, Bird HA, Conaghan PG. Assessment and management of neurogenic claudication associated with lumbar spinal stenosis in a UK primary care musculoskeletal service: a survey of current practice among physiotherapists. BMC musculoskeletal disorders. 2009;10:121 10.1186/1471-2474-10-121 19796387 PMC2762954

[5] Deyo RA, Mirza SK, Martin BI, Kreuter W, Goodman DC, Jarvik JG. Trends, major medical complications, and charges associated with surgery for lumbar spinal stenosis in older adults. JAMA. 2010;303(13):1259–65. 10.1001/jama.2010.338 20371784 PMC2885954

[6] Malmivaara A, Slatis P, Heliovaara M, Sainio P, Kinnunen H, Kankare J, et al Surgical or nonoperative treatment for lumbar spinal stenosis? A randomized controlled trial. Spine. 2007;32(1):1–8. Epub 2007/01/05. 10.1097/01.brs.0000251014.81875.6d . 17202885

[7] Weinstein JN, Tosteson TD, Lurie JD, Tosteson AN, Blood E, Hanscom B, et al Surgical versus nonsurgical therapy for lumbar spinal stenosis. N Engl J Med. 2008;358(8):794–810. Epub 2008/02/22. 10.1056/NEJMoa0707136 18287602 PMC2576513

[8] Tosteson AN, Lurie JD, Tosteson TD, Skinner JS, Herkowitz H, Albert T, et al Surgical treatment of spinal stenosis with and without degenerative spondylolisthesis: cost-effectiveness after 2 years. Ann Intern Med. 2008;149(12):845–53. Epub 2008/12/17. 10.7326/0003-4819-149-12-200812160-00003 19075203 PMC2658642

[9] Machado GC, Ferreira PH, Yoo RI, Harris IA, Pinheiro MB, Koes BW, et al Surgical options for lumbar spinal stenosis. Cochrane Database Syst Rev. 2016;11:CD012421 10.1002/14651858.CD012421 . 27801521 PMC6464992

[10] Fritsch CG, Ferreira ML, Maher CG, Herbert RD, Pinto RZ, Koes B, et al The clinical course of pain and disability following surgery for spinal stenosis: a systematic review and meta-analysis of cohort studies. Eur Spine J. 2017;26(2):324–35. 10.1007/s00586-016-4668-0 . 27443531

[11] McCaffery M, Beebe A. Pain: Clinical Manual for Nursing Practice: Mosby; 1989.

[12] Krebs EE, Carey TS, Weinberger M. Accuracy of the pain numeric rating scale as a screening test in primary care. J Gen Intern Med. 2007;22(10):1453–8. Epub 2007/08/02. 10.1007/s11606-007-0321-2 17668269 PMC2305860

[13] Zelman DC, Dukes E, Brandenburg N, Bostrom A, Gore M. Identification of cut-points for mild, moderate and severe pain due to diabetic peripheral neuropathy. Pain. 2005;115(1–2):29–36. Epub 2005/04/20. 10.1016/j.pain.2005.01.028 . 15836967

[14] Childs JD, Piva SR, Fritz JM. Responsiveness of the numeric pain rating scale in patients with low back pain. Spine. 2005;30:1331–5. 10.1097/01.brs.0000164099.92112.29 15928561

[15] Jensen MP, Turner JA, Romano JM. What is the maximum number of levels needed in pain intensity measurement? Pain. 1994;58:387–92. 10.1016/0304-3959(94)90133-3 7838588

[16] Ostelo RW, Deyo RA, Stratford P, Waddell G, Croft P, Von Korff M, et al Interpreting change scores for pain and functional status in low back pain: towards international consensus regarding minimal important change. Spine. 2008;33(1):90–4. Epub 2008/01/01. 10.1097/BRS.0b013e31815e3a10 . 18165753

[17] Fritz JM, Irrgang JJ. A comparison of a modified Oswestry disability questionnaire and the Quebec back pain disability scale. Phys Ther. 2001;81:776–88. 10.1093/ptj/81.2.776 11175676

[18] Fritz JM, Hebert J, Koppenhaver S, Parent E. Beyond minimally important change: defining a successful outcome of physical therapy for patients with low back pain. Spine. 2009;34(25):2803–9. Epub 2009/11/17. 10.1097/BRS.0b013e3181ae2bd4 . 19910868

[19] van Hooff ML, Mannion AF, Staub LP, Ostelo RW, Fairbank JC. Determination of the Oswestry Disability Index score equivalent to a "satisfactory symptom state" in patients undergoing surgery for degenerative disorders of the lumbar spine-a Spine Tango registry-based study. Spine J. 2016;16(10):1221–30. Epub 2016/06/28. 10.1016/j.spinee.2016.06.010 . 27343730

[20] Laursen B, Hoff E. Person-centered and variable-centered approaches to longitudinal data. Merrill-Palmer Quarterly. 2006;52(3):377–89.

[21] Nagin DS, Odgers CL. Group-based trajectory modeling in clinical research. Annu Rev Clin Psychol. 2010;6:109–38. Epub 2010/03/03. 10.1146/annurev.clinpsy.121208.131413 . 20192788

[22] Nagin D. Group-based modeling of development Cambridge, Mass: Harvard University Press,; 2005.

[23] Frankfurt S, Frazier P, Syed M, Jung KR. Using Group-Based Trajectory and Growth Mixture Modeling to Identify Classes of Change Trajectories. The Counseling Psychologist. 2016;44(5):622–60. 10.1177/0011000016658097 .

[24] Barry MJ, Edgman-Levitan S. Shared decision making—pinnacle of patient-centered care. N Engl J Med. 2012;366(9):780–1. Epub 2012/03/02. 10.1056/NEJMp1109283 . 22375967

[25] Hebert JJ, Fritz JM, Thackeray A, Koppenhaver SL, Teyhen D. Early multimodal rehabilitation following lumbar disc surgery: a randomised clinical trial comparing the effects of two exercise programmes on clinical outcome and lumbar multifidus muscle function. Br J Sports Med. 2015;49(2):100–6. Epub 2013/09/14. 10.1136/bjsports-2013-092402 . 24029724

[26] McGregor AH, Probyn K, Cro S, Dore CJ, Burton AK, Balague F, et al Rehabilitation following surgery for lumbar spinal stenosis. Cochrane Database Syst Rev. 2013;(12):CD009644 Epub 2013/12/11. 10.1002/14651858.CD009644.pub2 . 24323844 PMC11972841

