# Supplementary material for: Patients undergoing surgery for lumbar spinal stenosis experience unique courses of pain and disability: A group-based trajectory analysis
Source: PLoS One. 2019 Nov 7;14(11):e0224200. doi: 10.1371/journal.pone.0224200 (PMC6837529; doi:10.1371/journal.pone.0224200)
Supplement: S1 Table — Bayesian information criterion value closest to zero indicates best model fit 2 Minimum acceptable size at least 5% 3 Bayesian information criterion (for the total number of participants) 4 Bayesian information criterion (for the total number of observations). (DOCX) [file pone.0224200.s001.docx]

| S1 Table. Model selection results according to Bayesian information criterion and smallest group size^1^. | | | |
| --- | --- | --- | --- |
| Number of groups | Bayesian information criterion^2^ | Bayesian information criterion^3^ | Smallest group size^4^ |
| Leg pain outcome | | | |
| 1 | -4744.09 | -4746.80 | - |
| 2 | -4530.48 | -4535.22 | 49.5% |
| **3** | **-4484.75** | **-4492.21** | 14.4% |
| 4 | -4481.50 | -4491.67 | 2.5% |
| 5 | -4494.11 | -4507.01 | 12.8% |
| Back pain outcome | | |  |
| 1 | -4472.47 | -4475.19 | - |
| 2 | -4193.23 | -4198.66 | 49.7% |
| **3** | **-4152.29** | **-4159.07** | **13.1%** |
| 4 | -4158.51 | -4168.01 | 1.4% |
| 5 | -4157.26 | -4169.47 | 1.1% |
| Disability outcome | | |  |
| 1 | -8166.47 | -8169.11 | - |
| 2 | -7870.69 | -7870.69 | 44.9% |
| **3** | **-7808.23** | **-7799.66** | **29.1%** |
| 4 | -7831.28 | -7841.82 | <1% |
| 5 | -7830.21 | -7843.38 | <1% |
| ^1^ Bayesian information criterion value closest to zero indicates best model fit  ^2^ Minimum acceptable size at least 5%  ^3^ Bayesian information criterion (for the total number of participants)  ^4^ Bayesian information criterion (for the total number of observations) | | | |
