# Supplementary material for: Patients undergoing surgery for lumbar spinal stenosis experience unique courses of pain and disability: A group-based trajectory analysis
Source: PLoS One. 2019 Nov 7;14(11):e0224200. doi: 10.1371/journal.pone.0224200 (PMC6837529; doi:10.1371/journal.pone.0224200)
Supplement: S1 Fig — a1. sample 1 leg pain trajectories (N = 264), a2. sample 2 leg pain trajectories (N = 265); b1. sample 1 low back pain trajectories (N = 255), sample 2 low back pain trajectories (N = 255); c1. sample 1 disability trajectories (N = 259), c2. sample 2 disability trajectories (N = 260). Point estimates are average outcome scores (0–10 numeric pain rating scale or 0–100 modified Oswestry index). Dotted lines represent 95% confidence intervals. (DOCX) [file pone.0224200.s002.docx]

| 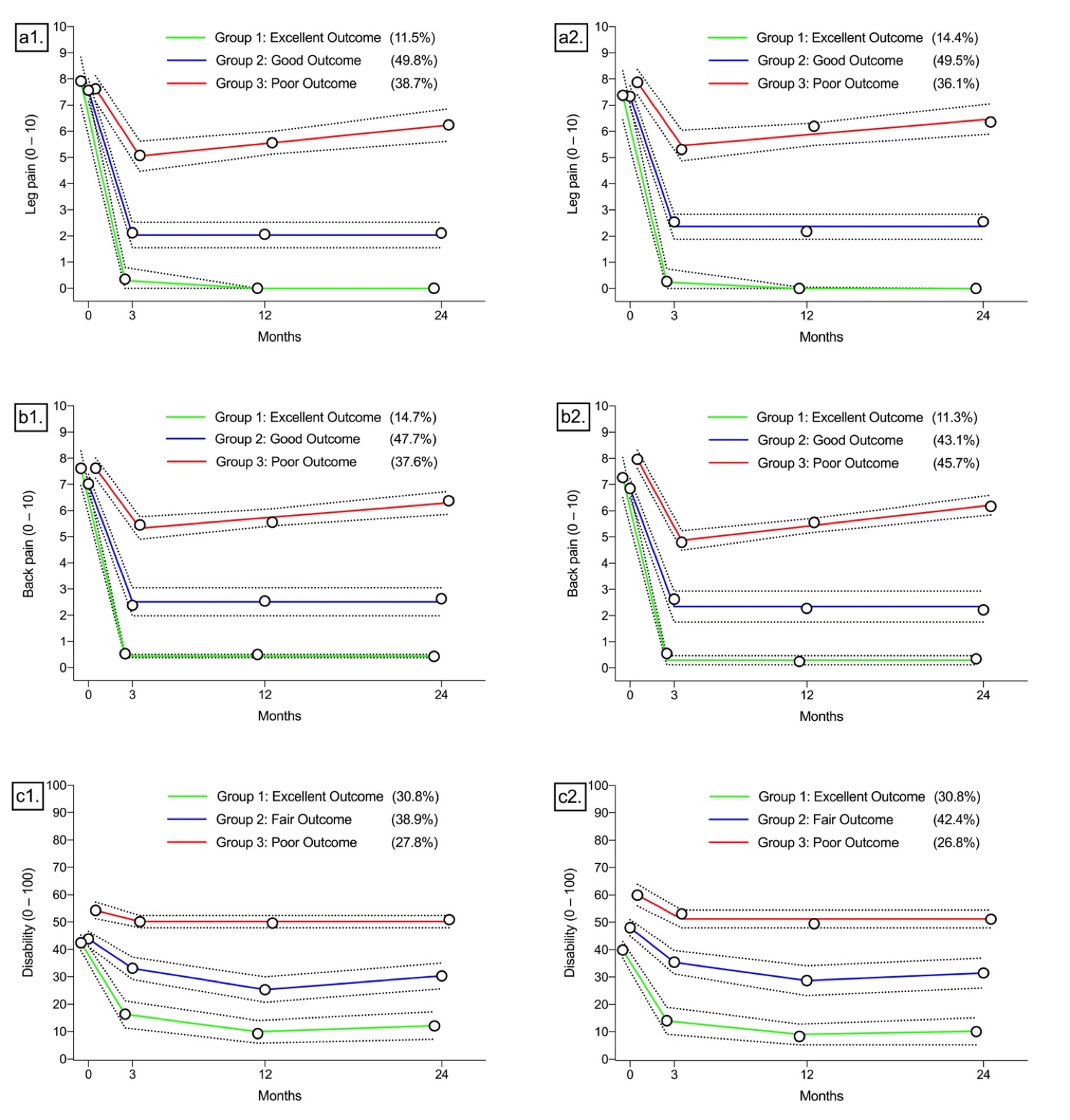 |
| --- |
| S1 Fig. Split sample clinical outcome trajectory groups with prevalence estimates.  a1. sample 1 leg pain trajectories (N = 264), a2. sample 2 leg pain trajectories (N = 265); b1. sample 1 low back pain trajectories (N = 255), sample 2 low back pain trajectories (N = 255); c1. sample 1 disability trajectories (N = 259), c2. sample 2 disability trajectories (N = 260). Point estimates are average outcome scores (0 – 10 numeric pain rating scale or 0 – 100 modified Oswestry index). Dotted lines represent 95% confidence intervals. |
